# Precision Medicine in Neurology: The Inspirational Paradigm of Complement Therapeutics

**DOI:** 10.3390/ph13110341

**Published:** 2020-10-26

**Authors:** Maria Gavriilaki, Vasilios K. Kimiskidis, Eleni Gavriilaki

**Affiliations:** 1 Postgraduate Course, School of Medicine, Aristotle University of Thessaloniki, 54124 Thessaloniki, Greece; kimiskid@auth.gr; 2 Laboratory of Clinical Neurophysiology, AHEPA Hospital, Aristotle University of Thessaloniki, 54621 Thessaloniki, Greece; 3 Hematology Department-BMT Unit, G. Papanicolaou Hospital, 57010 Thessaloniki, Greece; elenicelli@yahoo.gr

**Keywords:** complement activation, complement system proteins, nervous system diseases, neurodegenerative diseases, therapeutics, precision medicine

## Abstract

Precision medicine has emerged as a central element of healthcare science. Complement, a component of innate immunity known for centuries, has been implicated in the pathophysiology of numerous incurable neurological diseases, emerging as a potential therapeutic target and predictive biomarker. In parallel, the innovative application of the first complement inhibitor in clinical practice as an approved treatment of myasthenia gravis (MG) and neuromyelitis optica spectrum disorders (NMOSD) related with specific antibodies raised hope for the implementation of personalized therapies in detrimental neurological diseases. A thorough literature search was conducted through May 2020 at MEDLINE, EMBASE, Cochrane Library and ClinicalTrials.gov databases based on medical terms (MeSH)” complement system proteins” and “neurologic disease”. Complement’s role in pathophysiology, monitoring of disease activity and therapy has been investigated in MG, multiple sclerosis, NMOSD, spinal muscular atrophy, amyotrophic lateral sclerosis, Parkinson, Alzheimer, Huntington disease, Guillain–Barré syndrome, chronic inflammatory demyelinating polyneuropathy, stroke, and epilepsy. Given the complexity of complement diagnostics and therapeutics, this state-of-the-art review aims to provide a brief description of the complement system for the neurologist, an overview of novel complement inhibitors and updates of complement studies in a wide range of neurological disorders.

## 1. Introduction

Precision or personalized medicine was first introduced in 1999 prompted by the Human Genome Project [1]. Over the last decades, widely available genetic and functional assays have revolutionized diagnostic and therapeutic options across various disciplines [2]. Nevertheless, the lack of biopsy-driven studies of the nervous system, due to the uniqueness of this unapproachable tissue, impeded the understanding of the pathophysiological mechanisms involved in neurological diseases. Breakthrough advances in biology such as next-generation sequencing (NGS), the ability to identify potential cerebrospinal fluid (CSF) biomarkers or even develop 3D cell culture models of the human brain paved the way for the integration of precision medicine in neurology [3,4,5,6,7].

In parallel, the innovative application of the first complement inhibitor in clinical practice, eculizumab [8,9], gave rise to a renaissance of complement therapeutics [10]. Indeed, the latter has not only led to potentially more efficacious agents that overcome eculizumab’s limitations, some of which have gained FDA-approval and some are still undergoing clinical trials in phase I–III, but also to a novel classification of complement-mediated disorders, named as “complementopathies”. Complementopathies are characterized by complement dysregulation as a driver of disease pathogenesis and by therapeutic efficacy of complement inhibition [11]. The inspirational paradigm of complement therapeutics has facilitated the adoption of a precision medicine approach based on the individualized genetic profile of the patient in certain hematologic disorders [12]. Genetic variants of the gene encoding C5 have been associated with a poor response to eculizumab in patients with paroxysmal nocturnal hemoglobinuria (PNH) [13]. Although hematologic disorders have revolutionized this field, the number of complementopathies continues to increase across different specialties. During the last few decades, several studies identified elevated levels of complement components in the serum, CSF and biopsy specimens of patients with a wide range of neurologic disorders, paving the way for their use as predictive and prognostic biomarkers as well as therapeutic targets.

In view of the rapidly advancing knowledge in this field, the present state-of-the-art (describing the most recent and important data) review aims to provide a brief description of the complement system for the neurologist, an overview of novel complement inhibitors and updates of complement in a wide range of neurological disorders (myasthenia gravis, multiple sclerosis, neuromyelitis optica spectrum disorders, spinal muscular atrophy, amyotrophic lateral sclerosis, Parkinson, Alzheimer, Huntington disease, Guillain–Barré syndrome, chronic inflammatory demyelinating polyneuropathy, stroke and epilepsy).

A literature search was conducted through September 2020 of MEDLINE, EMBASE, Cochrane Library and ClinicalTrials.gov databases based on medical terms (MeSH) “complement system proteins”, “personalized medicine” and “neurologic disease”.

## 2. Complement for the Neurologist

The innate immune system is involved in both aging-related dysregulation of the central nervous system (CNS) and various neurodegenerative, demyelinating, inflammatory and cerebrovascular disorders [14]. In normal brain, complement proteins act as an immune surveillance system that plays a key role at synapse refinement and remodeling of neuronal connectivity [15]. Synapses tagged by complement are eliminated by microglial cells [16]. Moreover, developing reactive astrocytes induce the expression of complement proteins in the CNS [17]. This sensitive balance is preserved by the so-called neuroimmune regulators [18]. During aging of the brain, synapse degeneration is the hallmark of cognitive decline. Complement proteins and their receptors are implicated in the management of synapses’ engulfment by microglia in the developing, aged brain and also during disease [19,20]. Upregulated expression of C1q, the primary protein of the classical complement cascade up to 300-fold has been reported during normal aging of both animal models and human brain tissue [21]. The same applies for several neurodegenerative diseases [18]. For example, in Alzheimer’s disease (AD) excess C1q is found on synapses in the hippocampus of early disease animal models [22]. However, the signal that triggers synapse loss in aging and diseased brain is not well-understood.

Blood–brain barrier (BBB) generally restricts access of proteins to the brain. When complement is activated, its proteins may enter the brain in conditions that affect the BBB integrity [23], causing neurological damage. Such conditions may also release intracellular antigens leading to C1q recognition and complement activation, as we describe in the following paragraphs. Complement is also activated via C1q binding to antibodies that have neuronal antigens. Complement activation may also occur in physiological conditions of the brain, when BBB is intact [24]. However, the majority of the drugs that target the complement system are antibodies or proteins. Thus, access of these drugs to the CNS becomes a major issue when the BBB is mostly intact, as reviewed elsewhere [25].

The complement system consists of more than 30 proteins providing an important line of defense against pathogens [26,27]. The complement cascade is activated by the classical, alternative or lectin pathway. It should be noted, however, that the alternative pathway convertase accounts for approximately 80% of complement activation products in its role as an amplification loop [28].

The classical pathway is activated through C1q recognition of antibody–antigen complexes [29]. C1q also binds to pathogens and cell surface molecules that activate complement [30,31]. C1q C1q cleaves C1r, which activates C1s protease forming the C1 complex (i.e., C1qC1r2C1s2). Then, C4 and C2 are cleaved, leading to the formation of classical pathway C3 convertase (C4bC2a). The alternative pathway is continuously activated through spontaneous hydrolysis of C3, also known as “tickover” [32]. Activated C3(H_2_O) binds to factor B, that is then cleaved by factor D and generates the fluid-phase alternative pathway C3 convertase (C3(H_2_O)Bb) [33]. Following fluid-phase convertase formation, a surface bound convertase (i.e., C3bBb) is formed, traditionally depicted as the alternative pathway convertase. The lectin pathway is activated by mannose-binding lectins (MBLs) and other pattern recognition molecules, such as ficolins and collectin [34,35,36]. These molecules act via serine proteases (MASPs, mannose-associated serine proteases) that in turn generate the C3 convertase (C4bC2a). All three pathways lead to activation of terminal complement pathway. C5 convertases (i.e., C4b2bC3b and C3bBbC3b) cleave C5 into C5a and C5b. Then, C5b binds to C6 generating C5b-6 and then to C7, leading to C5b-7 [37]. Then, C5b-7 inserts into the membrane and binds C8 and C9 forming the membrane attack complex (MAC).

**Figure 1:**
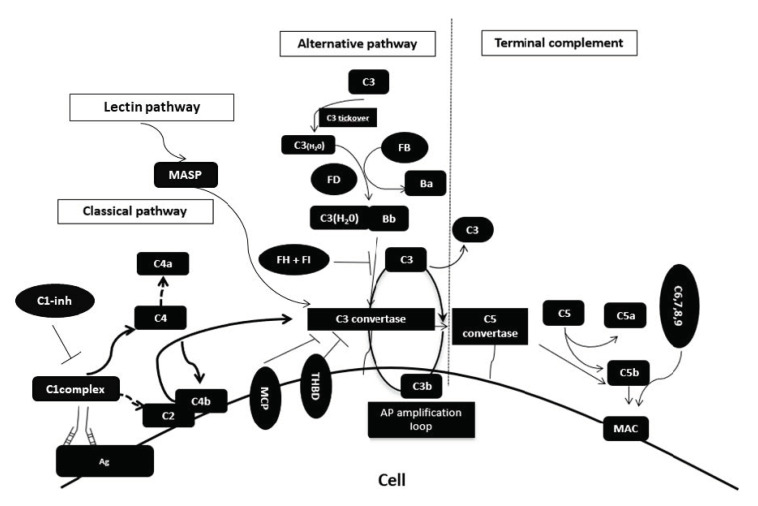
Elements of complement activation relevant with complement-mediated disorders.

Other pathways of complement activation are under investigation, primarily focusing on the role of the coagulation system [38,39]. Figure 1 summarizes key elements of complement activation relevant to complement-mediated disorders. Among them, numerous soluble and membrane-bound proteins regulate complement activation and play a major role in the pathogenesis of complement-mediated disorders (Figure 1).

The classical pathway is activated through C1q recognition of antibody–antigen complexes. C1q cleaves C1r, which activates C1s protease forming the C1 complex (i.e., C1qC1r2C1s2). Then, C4 and C2 are cleaved, leading to the formation of classical pathway C3 convertase (C4bC2a). During lectin pathway activation, mannose-associated serine proteases (MASPs) generate the C3 convertase (C4bC2a). The alternative pathway is continuously activated through spontaneous hydrolysis of C3, also known as “tickover”. Activated C3(H_2_O) binds to factor B, that is then cleaved by factor D and generates the alternative pathway C3 convertase. The alternative pathway of complement serves as an amplification loop for the lectin and classical pathway accounting for roughly 80% of complement activation products. In the presence of increased surface density of deposited C3b, the terminal complement is triggered, leading to membrane attack complex (MAC) formation on the surface of target cell. Complement pathway dysregulation results from loss-of-function mutations in regulatory factors (Factor H, I, THBD/thrombomodulin) and gain-of-function mutations (C3 and Factor B).

### 2.1. Novel Complement Inhibitors

Ongoing research for more than a century resulted in the discovery of the first-in-class complement inhibitor, eculizumab, a monoclonal antibody that blocks terminal complement by binding to C5. Eculizumab was initially approved for the treatment of PNH and atypical hemolytic uremic syndrome (aHUS) [8,9]. Eculizumab’s success into clinical practice renewed interest in complement therapeutics.

Nevertheless, current therapeutic agents are not able to inhibit complement activation, exclusively in the nervous system entailing the risk of systemic adverse events. Prolonged complement system inhibition could promote susceptibility to bacterial or viral infections, particularly meningococcal infections [40,41]. Thus, meningococcal vaccination is mandatory before therapy initiation [42]. However, eculizumab was approved as a treatment of PNH in 2007 and since then it has been involved in clinical trials of heterogeneous patient populations (patients with myasthenia gravis or NMOSD) [43]. No major adverse effects have been reported when appropriate monitoring and prophylaxis were applied.

A number of next-generation complement inhibitors are in the pipeline of clinical development for various complement-related disorders, including neurological disorders. Table 1 summarizes novel complement inhibitors under clinical development [44,45,46]. Further up-to-date details are described elsewhere [47]. Novel complement inhibitors aim to overcome eculizumab’s limitations. These primarily involve: (a) frequent intravenous infusions, (b) reduced efficacy in patients with certain mutations in C5, (c) breakthrough and (d) extravascular hemolysis [10].

A long-acting C5 inhibitor, ravulizumab, has recently gained approval for PNH treatment. Ravulizumab has the advantage of a longer half-life and subcutaneous administration [48,49]. In addition, it has shown sustained one-year safety and efficacy [50], as well as decreased breakthrough hemolysis [51]. Except for the route and frequency of administration, the specific complement pathway involved in a complement-mediated disorder and the potential infection risk are also to be considered for a personalized selection of a complement inhibitor.

### 2.2. Complement in Neurology of Infectious Diseases

In addition to neurodegenerative and neuroinflammatory diseases, the pathophysiology of various disorders with secondary neurologic manifestations has been postulated as complement mediated. Different complement activation pathways such as the lectin pathway in West Nile virus infection and activation of C3a and C5a in the CSF of patients with herpes simplex encephalitis have been involved in the uncontrolled inflammatory response during these infections [52,53]. Several studies highlighted the significance of complement activation in bacterial meningitis [54]. Interestingly, patients with genetic complement deficiencies and decreased levels of complement proteins in CSF presented with a favorable outcome [55]. Hence, the use of genetic profiling by the identification of patients with the genetic variation in the complement component five gene combined with the measurement of the anaphylatoxin C5a concentration in the CSF have been proposed as tools of a personalized approach in which we could predict who would benefit the most from treatment with C5 monoclonal antibodies in bacterial meningitis [56].

Nowadays, global efforts are made to fight coronavirus disease 2019 (COVID-19) pandemic and the complement system has been indicated as a promising target [57,58]. Neurologic symptoms have been reported in a notable proportion of COVID-19 patients in parallel with respiratory disease [59]. The exact pathophysiologic pathway of nervous system involvement in COVID-19 infection is poorly understood [60]. Yet, a possible mechanism might be through endothelial injury in several organs including the nervous system that resembles the phenotype of complement-mediated thrombotic microangiopathies [61]. Ongoing trials evaluate the efficacy and safety of vilobelimab, ravulizumab, narsoplimab, and AMY-101 in patients with severe COVID-19 [62,63,64].

## 3. Complement in Neurological Disorders

### 3.1. Myasthenia Gravis

Myasthenia gravis (MG) is an antibody-mediated autoimmune disease of the postsynaptic neuromuscular junction presenting with a fluctuating degree of weakness in ocular, bulbar, limb, and respiratory muscles. The pathogenesis of MG is one of the most thoroughly studied amongst neurological disorders and patients are expected to have a level of quality of life (QOL) comparable with the general population. However, 10–30% of MG patients prove to be refractory to conventional immunosuppression approaches [65].

#### 3.1.1. Complement Activation

The activation of complement via disease-specific autoantibodies to the acetylcholine receptor (AChR) has been known for over three decades [66]. Early animal studies of experimental autoimmune myasthenia gravis showed that complement factors aggregate at the end-plate of the neuromuscular junction [67]. Moreover, evidence from human studies demonstrated complement activation through changes in serum of MG patients as well as aggregation of the membrane attack complex in end-plates similar with animal models [68,69,70].

#### 3.1.2. Complement Inhibition

Data from both mice deficient in the complement regulators and direct complement inhibition suggests that therapeutic inhibition of MAC or other complement pathways could be beneficial in refractory MG [71,72,73,74]. However, eculizumab which inhibits complement activation through the C5 protein was only recently approved for use in treatment of anti-AChR antibody-positive refractory generalized myasthenia gravis (gMG) in adults [75]. Interestingly, eculizumab has shown long-term efficacy and improvements in MG-specific QOL outcome measures [76,77]. In addition, eculizumab showed remarkable benefits in real world data in an extended study population including patients with refractory gMG with myasthenic crisis, thymoma associated MG, and pregnancy [78]. Ongoing clinical trials are evaluating novel complement inhibitors as promising therapeutic targets (Table 2). Ravulizumab is now in phase 3 clinical trials for generalized MG [49]. Another C5 inhibitor, zilucoplan, demonstrated favorable outcomes in a phase II trial [79]. In the era of individualized therapeutics new consensus on MG treatment taking into consideration immune predictive biomarkers need to be reached.

### 3.2. Multiple Sclerosis and Neuromyelitis Optica Spectrum Disorder (NMOSD)

Multiple sclerosis (MS) is a chronic, inflammatory, demyelinating disease of the central nervous system that leads to variable axonal and neuronal damage [80]. Despite the fact that, MS has attracted scientific interest during the last few decades, the primary pathogenic event has not been discovered yet [81]. As a result, most of the disease modifying treatments aim to reduce clinical or radiological relapses which represent only the tip of the iceberg. The concept of resetting the immune system through autologous hematopoietic stem cell transplantation or certain novel pharmacological agents has gained ground over the last three decades [82]. However, MS remains incurable and different pathogenetic pathways such as the complement cascade are being explored mainly as diagnostic and therapeutic biomarkers in the context of personalized medicine [3].

The clinical distinction among MS and other demyelinating diseases of CNS such as Neuromyelitis Optica Spectrum Disorder (NMOSD) is challenging. Historically, NMOSD was thought to be a subtype of MS until the recognition of disease-specific aquaporin-4(AQP4)- immunoglobulin G (IgG) autoantibodies in 2004 [83]. Yet, 20% of patients are seronegative for AQP4 but a proportion of them express myelin oligodendrocyte glycoprotein (MOG)–IgG [84]. NMOSD has been traditionally considered an autoimmune inflammatory disease, treated mainly with immunosuppressive agents such as rituximab [85]. Scientific progress in the field is integrated in everyday clinical practice through continuously updated diagnostic and therapeutic guidelines [86,87,88].

#### Complement Activation/Inhibition

The role of complement pathway activation has been established in both relapsing remitting and progressive MS [89,90]. Depositions of C1q, C3d, and C5b-9 have been detected at the white matter lesions of MS [91]. Furthermore, complement proteins, activation products and inhibitors were found at MS plaques [92]. Recently, an animal study revealed an important therapeutic target for MS through genetic loss of the early complement pathway activation, specifically at the level of C3 [93]. The causal role of C3 in the pathophysiology of MS was further supported using a genetic association approach in humans. C3-rs2230199 coding variant was associated with both white and grey matter injury and cognitive impairment in MS patients [94]. Nevertheless, the specific target of those complement-mediated mechanisms has yet to be found. In the era of patient-tailored treatments, markers of complement activation have proven useful in identifying which patient could profit the most from acute treatment of MS relapses with apheresis therapy [95]. C3a in CSF at baseline assessment of patients with clinically isolated syndrome and newly diagnosed relapsing remitting MS could be a promising prognostic marker of disease activity given the fact that it was correlated with new lesions and “no evidence of disease activity”-3 status during follow-up [96]. Hence, complement markers might play a role for detecting disease activity, distinguishing MS from other demyelinating diseases and assessing response to treatment [97].

In contrast with MS, NMOSD complement inhibition has been studied in human trials. A recent study has shown complement-mediated death of neurons near astrocytes that was mitigated with complement inhibition [98]. A model indicating C1q activation by AQP4 autoantibodies in NMOSD was developed [99]. In this context, an animal studied showed that MOG-IgG induces mainly oligodendrocyte damage by activating complement [100]. Upregulation of the complement regulator CD55 has reduced NMOSD pathology [101]. Encouraging results were observed in a phase 2 study of eculizumab in 14 AQP4-IgG-positive patients showing the potential of the drug to prevent relapses [88]. These results have been confirmed in the most recent randomized, double-blind trial in 143 AQP4-IgG-positive patients [102]. It should be noted, however, that eculizumab did not improve measures of disability progression, suggesting that the long-term administration of eculizumab needs to be evaluated in view of the encouraging results of two additional clinical trials of immunotherapeutic agents in patients with NMOSD [103]. At present, direct complement inhibition is established as an FDA-approved NMOSD treatment and other C5 inhibitors such as ravulizumab are being tested (Table 2).

### 3.3. Neurodegenerative Diseases

#### 3.3.1. Spinal Muscular Atrophy

Spinal muscular atrophy (SMA) is a neuromuscular disease with an autosomal recessive pattern of inheritance leading to extensive motor neuron degeneration in the spinal cord and motor nuclei in the lower brainstem [104]. A single mutation of the survival motor neuron 1 (SMN1) gene, expressing the SMN protein, has been found in the majority of patients [105]. However, molecular malfunctions which lead to neuron degeneration following SMN protein deficiency are not fully understood. Until recently, the only available treatment was supportive care. The antisense oligonucleotide nusinersen and gene therapy with onasemnogene abeparvovec have been approved as disease-modifying therapies [106,107].

##### Complement Activation/Inhibition

Over the last decade, data implicated complement factor C1q in loss of motor neuron synapses in SMA [108]. In an SMA mouse model, classical pathway proteins C1q and C3 activation were upregulated contributing to microglia-mediated synapse elimination of spinal sensory–motor circuits [109]. The same study showed that pharmacological inhibition of C1q in vivo rescues valuable proprioceptive synapses and improves behavioral deficits in treated compared to SMA mice. Further research is needed to determine potential benefits of complement inhibition in SMA.

#### 3.3.2. Amyotrophic Lateral Sclerosis

Amyotrophic lateral sclerosis (ALS) is characterized by relentlessly progressive motor neuron degeneration of unknown pathogenesis that eventually leads to death. Despite the immense scientific interest and proposal of various potential pathogenetic pathways, no disease-modifying treatment (riluzole or edaravone) has been effective to reverse the course of the disease so far [110,111].

##### Complement Activation/Inhibition

An ongoing prospective case-control study, presented in Table 2, investigates the role of complement in ALS with the ambitious goal to find a target for therapeutic inhibition. The hypothesis of immune system’s involvement in ALS was confirmed many years ago [112]. Human studies proved the activation of the classical complement pathway and upregulation of C3 in the spinal cord, motor cortex and CSF of ALS patients [113,114,115,116]. Elevated levels of C5a and sC5b-9 were found in the serum of ALS patients suggesting that terminal complement components play the most important role [117]. Animal studies have shown that complement components’ levels may also serve as markers of disease progression [118]. Interestingly, in spite of the above-mentioned evidence of complement’s role in ALS, genetic deletion of the C1q, C3 gene in knockout mice models did not affect progression or survival in contrast with pharmacological antagonism of C5aR1 [119,120]. PMX205 is the antagonist of the C5a receptor and is currently undergoing development for future clinical trials in ALS.

#### 3.3.3. Alzheimer’s Disease

Alzheimer’s disease (AD) is the most common form of dementia affecting millions of older people worldwide [121]. The hallmarks of the disease pathophysiology comprise extracellular amyloid beta deposition and intracellular accumulation of hyperphosphorylated tau (p-tau) protein; yet genetic and environmental risk factors contribute to the complex mechanisms of aging and neurodegeneration [122,123,124]. The basis of AD management is still symptomatic, including treatment of behavioral changes, environmental manipulations, and caution regarding safety issues. Nevertheless, advances in the research of dementia promote a more pharmacologic approach. As a result, a trial of a cholinesterase inhibitor (donepezil, galantamine or rivastigmine) could be considered in patients with newly diagnosed AD.

##### Complement Activation/Inhibition

The aggregation of classical pathway’s proteins such as C1q, C4 and C3 in amyloid plaques in the cortex and the hippocampus of AD patients was confirmed by many studies in the early 1980’s [125]. Subsequent studies showed that the membrane attack complex, C5b-9, was also abundantly present in the AD cortex of post-mortem brain [126]. Thirty years later, the trigger for complement activation in AD is still unclear [127]. One theory suggests that amyloid β and tau aggregation may mediate microglial and astrocytic activation which stimulates complement cascade activation [128,129]. In support of this theory, infusion of anti-C1q antibody in tau transgenic mice led to a small but significant rescue of synapse density [130]. Antibody against C1q proved to efficiently prevent synapse loss in an AD animal model and has been successfully tested in a clinical trial (NCT03010046) for safety and tolerability in healthy volunteers [22,131]. Manipulation of complement activation through oral C5a receptor antagonist (PMX205) was also studied in AD mouse models resulting in significant reduction of amyloid deposition and glia activation [132]. Interestingly, some animal studies complicated more our understanding of complement role in AD by unravelling its neuroprotective actions [133]. The inhibition of C3 activation in the brain of human amyloid precursor protein transgenic mice lead to accumulation of degenerating neurons [134]. Nevertheless, genome wide association studies further indicated complement implication in AD pathogenesis by identifying single nucleotide polymorphisms (SNPs) correlating with risk of late-onset AD in genes encoding clusterin and CR1 [135]. Moreover, a whole-genome gene-expression profiling study showed that innate immunity together with microglia-related genes correlated best with late-onset AD [136].

In addition to their pathogenetic role, complement proteins have been studied as predictive biomarkers of mild cognitive impairment conversion to early AD with positive results [137,138]. Recent evidence suggests that astrocyte-and neuron-derived extracellular vesicles from AD patients effect complement-mediated neurotoxicity [139]. Taking the above-mentioned studies into consideration, evidence from clinical, animal and genetic research points toward complement as a promising key to unlock AD pathogenesis and treatment.

#### 3.3.4. Parkinson’s Disease

Parkinson’s disease (PD) is the most common cause of the clinical triad of resting tremor, rigidity and bradykinesia, the hallmark signs of parkinsonism. It has long been known that PD is characterized by dopamine deficiency at the basal ganglia and deposition of a-synuclein that forms Lewy bodies. However, the exact neuropathologic mechanisms remain elusive [140]. The pharmacologic, nonpharmacologic and surgical therapeutic armamentarium for the treatment of idiopathic PD is the broadest of any other neurodegenerative disease. An individualized approach is required based on each patient’s age, symptoms, disease severity, disability status, and degree of physical activity.

##### Complement Activation/Inhibition

Even though animal models are particularly useful in translational research, the ideal PD mouse model has not yet been developed [141]. In vitro PD studies correlated complement activation with a-synuclein and Lewy bodies [142]. Human studies showed controversial results; some confirmed the involvement of the classical complement pathway by recognizing anti C3d, C4d, C7 and C9 antibodies in substantia nigra or by the aggregation of iC3b and C9 in Lewy bodies of PD patients which were not present in controls [143,144]. Yet, another study failed to correlate complement activation with cortical Lewy Bodies [145]. There is emerging evidence that microglia is leading to neuronal death through the C1q-mediated pathway [146]. In search for biomarkers, complement proteins have been identified in the serum and CSF of PD patients [147,148]. In particular, clusterin and complement C1r were decreased compared to controls and have been proposed as useful biomarkers of disease progression [149]. A recent proteomic analysis of serum exosomes of PD patients found microglial C1q significantly decreased in PD and suggested a complex function of C1q in its pathophysiological mechanism [150].

Animal studies aiming to improve parkinsonism through genetic deletion of C1q and C3 failed [151,152]. This could be explained by the fact that complement receptor 3 (CR3) was found to be neuroprotective against dopaminergic neurodegeneration in a CR3 knockout mice model [153]. To sum up, it is urgent to understand complement involvement and inhibition thoroughly in human studies, as there is accumulating evidence of complement dysregulation in PD pathogenesis.

#### 3.3.5. Huntington’s Disease

Huntington’s disease (HD) is an autosomal dominant neurodegenerative disorder caused by a cytosine-adenine-guanine (CAG) trinucleotide repeat expansion in the huntingtin gene [154]. Its clinical characteristics (chorea, psychiatric illness, and dementia) are thought to be the end-result of the toxic effect of the mutant huntingtin protein to brain cells [155]. As the pathophysiology is still not well-understood, treatment remains supportive [156].

##### Complement Activation/Inhibition

Clinical studies indicated that complement was activated and in fact produced locally by reactive microglia in post-mortem brain tissue of patients with HD in contrast to normal brain [157]. Moreover, microglia activation correlated with clinical severity in human positron-emission tomography studies of HD [158,159]. Regarding the question of whether innate immune dysregulation is a direct consequence of mutant huntingtin or a secondary event, an animal study concluded that microglia has impaired migration responses to C5a from early postnatal HD transgenic mice [160]. A human study searching for possible biomarkers in plasma and CSF showed that complement components C7 and C9 were significantly upregulated in HD patients compared to controls and that increasing levels of clusterin were associated with disease progression [161]. In line with these data, animal studies confirmed that inhibition of C5a receptor using PMX53 and PMX205 markedly inhibited neurodegeneration [162]. However, in another transgenic mouse model of HD characterized by overexpression of the human huntingtin gene, genetic deficiency in C3 could not affect disease progression similarly to an ALS and PD model [119,151,163]. Recently, another target in the complement cascade (C5aR2) was investigated in animal studies and its genetic deletion resulted in improved motor and cognitive performances [164].

### 3.4. Immune-Mediated Polyneuropathies

Guillain–Barré Syndrome (GBS) is characterized by acute immune-mediated polyneuropathies which cause a rapidly evolving, monophasic paralyzing disease usually following an acute infection [165]. Chronic inflammatory demyelinating polyneuropathy (CIDP) is a relapsing-remitting or progressive immune-mediated polyneuropathy primarily affecting adults [166]. For many decades, the only available therapy for both has been corticosteroids, intravenous immunoglobulin or plasma exchange [167,168]. It is estimated that almost 20% of GBS patients treated with these immune therapies still present some disability after 6 months [169].

#### 3.4.1. Complement Activation

Early studies in autopsies of GBS patients have shown terminal complement activation and MAC deposition on Schwann cells [170]. Subsequently, human studies showed that complement components C3a and C5a were significantly elevated in the CSF of patients suffering from GBS [171]. In addition, IgG anti-ganglioside antibodies that lead to GBS cause complement-mediated disruption of interactions between Schwann cells and axons [172,173].

Regarding CIDP, treatment-naïve patients had elevated serum and CSF levels of soluble terminal complement complex (sTCC) and the increase was correlated with clinical disability [174]. On the contrary, patients with spontaneous CIDP remission presented with decreased complement activation markers over time [175]. However, the therapeutic efficacy of IVIg was not related to C3a, C5a or soluble MAC levels.

#### 3.4.2. Complement Inhibition

Terminal complement inhibition by eculizumab prevented neuropathy caused by anti-ganglioside antibodies in a murine model [176]. This evidence prompted the first, small-scale clinical trial of eculizumab that did not however reach its primary end-point [177]. Similarly, a randomized double-blind placebo-controlled phase 2 trial with 34 participants [176,177,178] failed to reach the predefined response rate [178]. There is an ongoing phase II clinical trial (Table 2), investigating the safety and tolerability of C1q inhibition when administered in combination with IVIg in GBS patients. Except for the patient population, the significant issues of combination treatment and dosage modifications need to be further addressed by future studies. Currently, no study has investigated the therapeutic potential of complement inhibition in CIDP.

### 3.5. Cerebrovascular Disease

Cerebrovascular disease comprises ischemic stroke, intracranial and subarachnoid hemorrhage with the majority of events caused by cerebral infarction [179]. Despite the fact that patients presenting with an ischemic stroke undergo pharmacological and mechanical reperfusion therapy, stroke remains a leading cause of disability or even death [180,181].

#### 3.5.1. Complement Activation

Neuroinflammation mainly induced by activation of the complement cascade has been correlated with infarct development and post-reperfusion tissue injury both in human and animal models [182,183,184,185]. Most data incriminate C3a and C5a as critical determinants of brain inflammation [186,187,188]. At the same time, there is evidence that C3a is selectively protective against neuronal death [189]. Accumulating evidence incriminates the lectin pathway in post-stroke injury considering the correlation of mannose-binding lectin (MBL) deficiency with better outcomes [190]. Interestingly, the terminal soluble C5b-9 or MAC is activated even after 12 days during post-stroke recovery [191]. Recent human data suggest that plasma C3 and C3a levels may be not only a diagnostic but also a predictive biomarker of stroke outcome [192].

Complement cascade activation has been also observed in hemorrhagic stroke [193]. In humans suffering from subarachnoid hemorrhage, CSF and plasma C3a, C4a levels were significantly elevated and related with worse outcomes [194]. In addition to these, a recent study showed that C5 SNP correlated with poor outcomes in patients suffering from aneurysmal subarachnoid hemorrhage (SAH) [195].

#### 3.5.2. Complement Inhibition

Despite the above-mentioned body of evidence, there is currently no clinically relevant anti-complement or even anti-inflammatory strategy for stroke therapy. At the moment, the thrombolytic agent tissue plasminogen activator (tPA) is the only FDA-approved medication, but its use is limited due to a narrow therapeutic time window. Targeted complement inhibition at ischemic mice brain prevented microglial activation and local phagocytosis and exhibited a prolonged (24 h) therapeutic window [196]. Following the observation that C3a and C5a influences post-ischemic inflammation, a number of studies proved that C3a and C5a receptor antagonism improves the repair of reperfused brain [197,198]. Moreover, C1 inhibition was neuroprotective in brain ischemia and in a reperfusion injury model independently of C1q deficiency [199]. In this context, C3 inhibition improved the safety, efficacy and therapeutic window of recanalization therapy through reduced haemorrhagic transformation and resulted in better cognitive outcomes [200]. The hypothesis of a lectin-driven mechanism was supported by studies involving the administration of MBL inhibitors up to 18 h after ischemia that reported improved neurological outcome [201]. The same applied for inhibitors of the alternative pathways in mice [202].

Similarly, C3a and C5a inhibition reduced inflammation and brain edema after intracerebral hemorrhage in a mouse model [203,204]. A recent study added an extra clue about the possible benefit of C5 inhibition in a mouse model of SAH by observing less activated microglia and apoptosis in mice lacking the C5a receptor or treated with C5-specific antibodies [195].

### 3.6. Epilepsy

Epilepsy is a chronic brain disease characterized by an enduring predisposition to generate epileptic seizures [205]. The exact pathophysiological mechanisms that lead to recurrence of epileptic seizures remain unknown. Hence, management is based on controlling seizures while avoiding pharmaceutical side effects. Although a significant proportion of epileptic patients can be treated satisfactorily with currently available therapeutic means, 30–40% continue to suffer from uncontrollable epileptic seizures and their multifarious consequences. The identification of the pathogenic process underlying epileptogenesis and ictogenesis would enable the development of predictive biomarkers and new therapeutic targets that may reduce the above-mentioned epileptic burden.

#### 3.6.1. Complement Activation

Neuroinflammation with the participation of the complement cascade is thought to be part of the epileptic pathophysiologic process in both humans and animal models [206,207]. Twenty-five years ago, Basaran et al. demonstrated that the concentration of serum C3 in untreated patients was significantly higher than in healthy controls [208]. The contribution of the complement cascade in rat models of temporal lobe epilepsy (TLE) has been documented by the cytotoxicity of C5b6, C7, C8, and C9 infusion into the hippocampus of rats leading to electrographic seizures [209]. In patients with drug-resistant TLE, an elevated expression of C1q and C3d has been consistently found [210,211]. Recent studies reported hyperactivation of the complement pathway both in patients with epilepsy compared to healthy controls and in untreated patients compared to treated [212]. Lately, it has been shown that phagocytic signaling with complement participation occurs in human refractory epilepsy [213]. Regarding status epilepticus (SE), it has been suggested that enhanced activation of the classical complement pathway may play a crucial pathogenic role [214].

#### 3.6.2. Complement Inhibition

Complement-mediated inflammation may be part of the pathophysiological processes underlying epilepsy, but its therapeutic potential has not been explored so far. In murine models, the administration of C5ar1 antagonist exerted anticonvulsant activity indicating a potential anti-epileptic drug for pharmaco-resistant patients [215]. A recent study in an SE rat model investigated the effect of acute treatment with C1 esterase inhibitor on the prevention of SE-induced learning and memory deficits [216]. The results suggest that complement inhibition may have a protective role against cell death but does not mitigate the learning and memory deficits of SE model rats. Currently, there are no ongoing clinical trials testing complement inhibition in epilepsy [217].

## 4. Conclusions and Future Perspectives

Over the last few years, accumulating evidence has implicated complement in a variety of neurological diseases with different pathophysiology. A number of these primary nervous system disorders remain incurable with significant negative consequences on the quality of life of patients and their families. The advent of complement therapeutics resulted in numerous recent and ongoing clinical trials in the field of neurology. Although complement proteins have been recognized to be involved in the pathophysiology of a wide variety of neuroinflammatory and neurodegenerative diseases, the efficacy of complement inhibitors to treat some of these diseases has been promising. Already, complement inhibition is an approved therapy for MG and NMOSD related with specific antibodies. The common denominator in both disorders is a circulating autoantibody (AChR and AQP4 antibodies) which mediates complement activation and subsequent tissue damage. Advances in the field contribute to our understanding of complement system involvement and also raise hope for a tailored approach in neurodegenerative disorders. Although complement inhibition is unlikely to reverse disease progression in MS, AD or PD, it could modify GBS, CIDP or infectious disease course. Inevitably, different diseases mechanisms require different therapeutic approaches which target alternative parts of complement cascade that are activated in each case and will require different routes of administration.

Beyond their use as therapeutic targets, proteins at different levels of the complement cascade in blood, CSF and tissues could become informative biomarkers which would direct the selection of the right therapeutic approach for the right patient and could monitor disease progression and therapeutic efficacy. Indeed, complement activation at the level of C3 seems to be a pivotal marker of MS disease activity as well as a therapeutic target. Moreover, terminal complement complex has been proved to be a useful predictive biomarker of mild cognitive impairment conversion to early AD. As a result, complement could pave the way for a personalized approach to the therapeutic management and monitoring of these disorders.

Furthermore, it is now clear that animal models may not replicate human brain mechanisms in health and disease sufficiently enough to be a useful guide for further clinical trials [218]. Ongoing research is urgently needed to address the following questions. It is of paramount importance to develop models which could better depict brain diseases as well as identify biomarkers predicting which patient might benefit from anti-complement therapy.

1. Is complement a driver or an innocent bystander in neurological disorders?
2. Will novel complement inhibitors overcome the obstacle of BBB and CNS accessibility?
3. Will specific inhibitors of complement pathways be safe and effective? Is there a way to keep the balance between the neuroprotective and detrimental roles of complement in CNS?
4. Are there tools for diagnosis and monitoring of patients that will benefit from complement inhibition?

The growing evidence of complement involvement in numerous neurological disorders along with the development of new complement inhibitors raise hope for future implementation of a precision medicine approach in the management of these detrimental diseases.

## Figures and Tables

**Table 1:** Summary of novel complement inhibitors under clinical development.

Inhibitor	Target
Eculizumab	C5
Ravulizumab	
ABP959	
SKY59/RO7112689	
Tesidolumab	
REGN3918	
Mubodina	
Coversin	
Zilucoplan	
Cemdisiran	
Avacincaptad pego	
AMY-101	C3
APL-2	
mini-FH/AMY-201	AP C3 convertase
LNP023	Factor B
IONIS-FB-LRx	
Danicopan	Factor D Factor D
Lampalizumab	
CLG561	properdin
Sutimlimab	C1s
Narsoplimab	MASP-2
Mirococept	C3/C5 convertases
Avacopan	C5aR1
PMX205	
IFX-1	C5a

AP: alternative pathway; MASP: mannose-associated serine protease.

**Table 2:** Summary of ongoing clinical trials investigating complement therapeutic targets and the role of the complement system in neurologic disorders.

Title	Aims and Interventions	Phase/ Study Type	Trial Number
*Myasthenia Gravis*			
A Phase 3, Randomized, Double-Blind, Placebo-Controlled, Multicenter Study to Evaluate the Safety and Efficacy of Ravulizumab in Complement-Inhibitor-Naïve Adult Patients with Generalized Myasthenia Gravis	To compare the safety and efficacy of ravulizumab versus placebo for the treatment of complement-inhibitor-naïve adult participants with generalized Myasthenia Gravis	III	NCT03920293
A Phase 3, Multicenter, Randomized, Double Blind, Placebo-Controlled Study to Confirm the Safety, Tolerability, and Efficacy of Zilucoplan in Subjects with Generalized Myasthenia Gravis	To confirm the efficacy, safety, and tolerability of zilucoplan versus placebo in subjects with generalized Myasthenia Gravis	III	NCT04115293
An Open-Label, Multicenter Study to Evaluate the Efficacy, Safety, Pharmacokinetics, and Pharmacodynamics of Eculizumab in Pediatric Patients with Refractory Generalized Myasthenia Gravis (gMG)	To evaluate the efficacy, safety, pharmacokinetics, and pharmacodynamics of eculizumab in the treatment of pediatric refractory gMG based on change from Baseline in the Quantitative Myasthenia Gravis score for disease severity.	III	NCT03759366
*Neuromyelitis optica spectrum disorders (NMOSD)*			
A phase 3, external placebo-controlled, open-label, multicenter study to evaluate the efficacy and safety of ravulizumab in adult patients with Neuromyelitis Optica Spectrum Disorder (NMOSD)	To evaluate the efficacy as measured by time to first relapse and safety through treatment-emergent adverse events of ravulizumab for the treatment of adult participants with NMOSD	III	NCT04201262
A Phase 2/3 Open-Label, Single-Arm Trial to Evaluate the Safety and Activity of Eculizumab in Pediatric Patients with Relapsing NMOSD	To study the safety and efficacy of eculizumab in pediatric participants with relapsing NMOSD	II/III	NCT04155424
A Phase III, Open-label, Extension Trial of ECU-NMO-301 to Evaluate the Safety and Efficacy of Eculizumab in Patients with Relapsing Neuromyelitis Optica (NMO)	To evaluate the long-term safety and efficacy of eculizumab in subjects with relapsing NMO who have completed the initial double-blind, randomized, placebo-controlled trial ECU-NMO-301.	III	NCT02003144
*Guillain–Barré Syndrome (GBS)*			
A Clinical Study of ANX005 and IVIG in Subjects with Guillain Barré Syndrome (GBS)	Safety and tolerability of ANX005 when administered in combination with IVIg in GBS	I-II	NCT04035135
*Amyotrophic Lateral Sclerosis (ALS)*			
Amyotrophic Lateral Sclerosis and the Innate Immune System	To investigate the role of the innate immune system, and especially the complement system, in patients with ALS	Case- Control Observational Study	NCT02869048
*Cerebrovascular Disease*			
Evaluation of Lectin Pathway in Assessment of Unstable Carotid Plaque	To evaluate the possible role of lectin pathway in affecting stability of carotid atherosclerotic plaques and the possible correlations with clinical neurologic features	Prospective Cohort	NCT03822195
STRATifying Risk for intracErebral haemorrhaGe and Neurodevelopmental DIsorders in Newborns	To investigate the role of gestational age on the prevalence of coagulation factors and components of the complement system in neonates and their role for the development of brain hemorrhage.	Non- Randomized clinical trial	NCT04140812
